# 

**Published:** 1908-08-22

**Authors:** 


CONTENTS.

| | PAGE |
|---|---|
| **Conservative Surgery** | 537 |
| **The Poisons and Pharmacy Bill** | 538 |
| **Annotations**— | |
| Cosmopolitanism—The Duties of "Dressers"—School Hygiene | 539 |
| **Medical Opinion and Movement** | 540 |
| **Hospital Clinics**— | |
| The Psychology of Failure and Success. By T. Claye Shaw, M.D., F.R.C.P. | 543 |
| **Special Article**— | |
| Venomous Caterpillars | 545 |
| **Medicine**— | |
| Myocarditis and Myocardial Disease—III. | 547 |
| Enlargements of the Spleen—V. | 548 |
| **Surgery**— | |
| Œsophageal Obstruction.—III. | 549 |
| Epulis | 550 |
| **Practical Notes on Diagnosis and Treatment** | 550 |
| **Life Assurance**— | |
| Practical Hints upon Medical Examination for Life Assurance.—III. | 551 |
| **Obstetrics**— | |
| Labour in Minor Degrees of Pelvic Contraction | 552 |
| **The Royal Army Medical Corps Section**— | |
| The Organisation of a Territorial Army General Hospital | 553 |
| **Public Health and Hygiene**— | |
| Notes on Medical Inspection of School Children | 554 |
| **Therapeutics**— | |
| Prescriptions and Dispensing | 555 |
| **The General Practitioner's Column**— | |
| Useful Methods of Diagnosis and Treatment | 556 |
| **Hospital Administration**— | |
| Graduate Study on the Continent—Germany | 557 |
| **Nursing Administration**— | |
| The Domestic Staff | 559 |
| **Editor's Letter-Box** | 560 |
| **New Appliances and Things Medical** | 560 |
| **News and Coming Events** | 561 |

